# Comparative analysis of soil organic carbon across different land types in plateau wetlands using Kriging interpolation based on spatial heterogeneity

**DOI:** 10.1371/journal.pone.0328246

**Published:** 2025-07-23

**Authors:** Ximei Wen, Wenmin Luo, Xiuyuan Yang, Fupeng Li, Zhenming Zhang

**Affiliations:** 1 Guizhou Institute of Mountainous Resources, Guizhou Academy of Sciences, Guiyang, Guizhou, People’s Republic of China; 2 Guizhou Institute of Biology, Guizhou Academy of Sciences, Guiyang, China; 3 College of Resources and Environmental Engineering, Guizhou University, Guiyang, China; Sathyambama Institute of Science and Technology: Sathyabama Institute of Science and Technology (Deemed to be University), INDIA

## Abstract

Wetlands, as an important global carbon reservoir, contribute significantly to the terrestrial carbon cycle. However, the high spatial heterogeneity of wetland ecosystems poses a considerable challenge to accurate estimation and mapping of soil organic carbon (SOC). In this study, we focused on Caohai Wetland in Guizhou Province, China, a typical plateau freshwater wetland, to evaluate the spatial variability of SOC across five land use types. A total of 122 surface soil samples were collected, and SOC content was analyzed using three Kriging interpolation methods—Ordinary Kriging (OK), Simple Kriging (SK), and Universal Kriging (UK)—in combination with four semi-variogram models (Gaussian, Hole effect, J-Bessel, and K-Bessel). The results indicated that SOC distribution varied significantly among different soil types. The spatial variability was highest in swamp and grassland soils and lowest in agricultural and forest soils. Among the semi-variogram models, the J-Bessel model showed the best performance in capturing local variation patterns. OK and SK yielded lower RMSE values (2.41) and higher R² (0.913 and 0.911, respectively) than UK (RMSE = 2.80; R² = 0.863). Principal component analysis revealed that SOC was positively correlated with total nitrogen, available nitrogen, Cd, Zn, DDT, and OCPs, and negatively correlated with pH. The cumulative variance explained by the two principal components was 81.3%. These findings demonstrate that Bessel-type models combined with Ordinary or Simple Kriging provide superior prediction accuracy in highly heterogeneous wetland soils. The methodology offers a scientific basis for SOC spatial modeling and targeted soil carbon management strategies in plateau wetland ecosystems.

## 1. Introduction

Wetlands, known as the “Kidneys of the Earth,” are one of the four major terrestrial ecosystems and play an important role in mitigating climate change through carbon sequestration [1]. Studies have shown soil carbon storage is more than three times that of atmospheric carbon storage [2,3]. However, ongoing climate change and anthropogenic land-use pressures have altered wetland structure and function, leading to increased interest in assessing their spatial carbon distribution and stability. However, ongoing climate change and anthropogenic land-use pressures have altered wetland structure and function, leading to increased interest in assessing their spatial carbon distribution and stability.

Soil organic carbon (SOC), derived from plant residues, microbial biomass, and humus, is a key indicator of soil quality and ecosystem health [4,5]. The spatial distribution of SOC is known to be influenced by multiple interacting factors such as land use, soil texture, moisture, topography, and contamination [6,7]. In wetland landscapes, where land cover is spatially heterogeneous and microtopography is variable, SOC often exhibits strong spatial autocorrelation and discontinuity. As a result, modeling SOC spatial variability in such ecosystems is particularly challenging.

Geostatistical interpolation methods, especially Kriging, are commonly used to predict soil properties in unsampled locations. Kriging offers unbiased and optimal spatial predictions by accounting for spatial autocorrelation among samples [8]. While Ordinary Kriging (OK) remains the most widely applied, alternative methods such as Simple Kriging (SK) and Universal Kriging (UK) are also used, depending on data assumptions and underlying spatial trends [9]. In addition, variogram model selection (e.g., Gaussian, Hole effect, Bessel-type) critically affects interpolation performance, particularly in heterogeneous environments.

However, few studies have systematically compared different Kriging methods combined with multiple variogram models to evaluate their relative effectiveness in predicting SOC under complex wetland conditions. Most past research focuses on a single interpolation approach or lacks a quantitative evaluation of model accuracy [10,11].

In this context, the Caohai Wetland in Guizhou Province—a representative plateau freshwater wetland—was selected as the study area. This research aims to (1) evaluate the spatial distribution of SOC across land use types using field sampling data; (2) compare the performance of three Kriging interpolation methods under four variogram models in predicting SOC; and (3) identify the key environmental variables influencing SOC variability. The results will provide a reference for accurate spatial mapping of SOC in plateau wetlands and support science-based strategies for carbon management in ecologically sensitive areas.

## 2. Materials and methods

### 2.1. Study area

**Fig 1:**
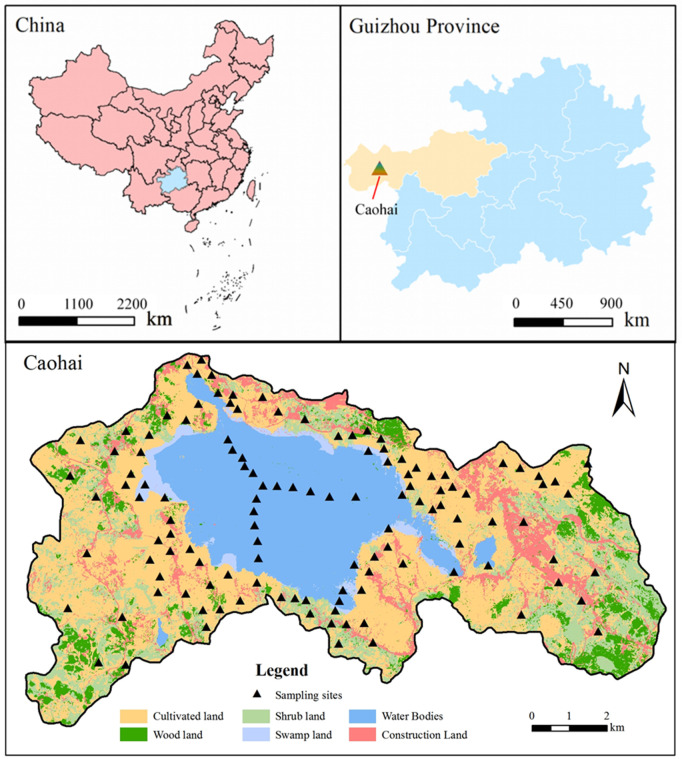
Geographic location of the study area.

Caohai (26°45′N ~ 27°00′N, 104°10′E ~ 104°25′E) is in the hinterland of Wumeng Mountain in the central tip of China’s Yunnan-Guizhou Plateau and is a typical freshwater wetland located on a plateau ecosystem at the same latitude in the world. The total area of Caohai National Nature Reserve is about 120 km^2^, with an elevation of 2171.7 m, and the lake area is 31km^2^, with a water depth of up to 9 m. It has a subtropical monsoon climate, with an annual average temperature of about 10.6°C, with the highest temperature reaching 36.8°C, and the lowest temperature reaching 5°C [12]. The Caohai Basin as a whole has a high altitude, low temperature and relatively high humidity, and the soil type is mainly plateau yellow-brown loam, with a pH value of 5.0–6.0 and strong soil leaching [13]. Around the lake, lake swamp soils are developed. Most of the lakeshore areas above the standing water level are developed as dry soils for growing crops. In some areas of lake marsh with low relief, it has also been gradually transformed into cultivated land with a cover distribution of woodland, cropland, grassland and mudflat centered on the watershed from outside to inside [14,15]. The geographical location of the Caohai is shown in Fig 1.

### 2.2 Sample collection and processing

Soil and surface sediments of different land use modes (grassland, agricultural land, forest land and marshland) around the Caohai in Guizhou Province were sampled. The surface sediment sampling routes were designed to take the center of the Caohai as the starting point, and then radiate in three directions, with sampling points in the eastern, southwestern and northwestern areas of the Caohai. Sampling points were distributed across five land use types including sediment, swamp, grassland, cropland, and woodland. A total of 122 surface soil samples (0–20 cm) were collected using a stainless-steel auger, and approximately 500 g of soil was collected per point. Sampling locations were georeferenced using a handheld GPS (accuracy ±3 m), and the spatial distribution is shown in Fig 1. The collected soil samples were transported back to the laboratory to remove debris such as gravel and residual roots, and then placed in a cool and ventilated place to dry naturally. After drying, the soil samples were mixed thoroughly and half of them were divided according to the diagonal quadratic soil sampling method to be kept as spare samples, and the rest of the samples were sieved through holes for the determination of their properties.

### 2.3. sample testing

pH is measured using the potentiometric method; organic carbon is measured using the potassium dichromate external heating method; total nitrogen is measured using the Kjeldahl method and a fully automatic nitrogen analyzer; total phosphorus is measured using the acid-soluble-antimony-antimony method; available phosphorus is measured using hydrochloric acid and ammonium fluoride extraction and the molybdenum-antimony anti-colorimetric method; alkali-soluble nitrogen is measured using the alkali-soluble diffusion method.

### 2.4. Semi-variational function fitting and Spatial Interpolation

The semi-variance function is a function between the semi-variance value γ(x, h) and the distance. where the semi-variance value represents the spatial correlation and is equal to the expectation of the difference between the thickness values at distance h in all sampling points. The experimental semi-variance function is usually characterized by three parameters: (1) the nugget value, which is mainly due to spatial variability and measurement errors. According to Cambardella et al. (1994) given criteria, the nugget-base ratio determines the spatial dependence of soil properties, where nugget-base ratios <25%, 25–75%, and >75% represent strong, moderate, and weak spatial dependence, respectively; (2) abutment values, which in general characterize the maximum variance between pairs of data; and (3) variance ranges, which represent the furthest distance at which there exists a correlation in the soil property parameters [16]. The experimental variability function is a discontinuous distribution function obtained from the collected data, and the theoretical variability function needs to be used to fit the experimental variability function to obtain a continuous variability evaluation. In this study, we sampled four fitting models, namely, Gaussian model, null effect, K-Bessel and J-Bessel, for the prediction of SOC. The expressions of the four functions are shown in S1 Table in S1 File [17].

Model selection was guided by the spatial patterns observed in exploratory data analysis and the ecological characteristics of the wetland landscape. The Bessel models were expected to capture local patchiness and short-range variability due to oscillatory spatial patterns. Model performance was assessed based on root mean square error (RMSE), mean error (ME), and coefficient of determination (R²) through cross-validation. Details of the empirical and fitted semi-variograms are illustrated in Fig 3 and Table 2.

**Fig 2:**
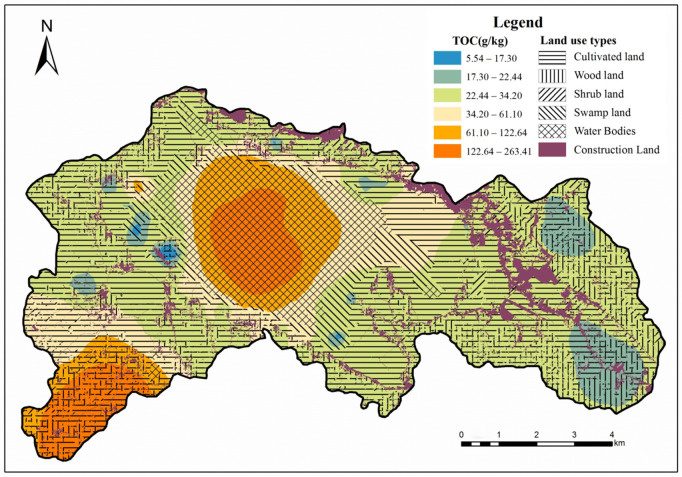
Spatial distribution of organic carbon in different types of soils in the Caohai.

### 2.5. Spatial interpolation methods

Kriging is a method of assuming distances or directions between sample points, reflecting spatial correlation, that can be used to explain variations in surfaces. Kriging tools fit a mathematical function to a specified number of points or to all points within a specified radius to determine the output value at each location (ESRI). A commonly used method for spatial prediction of regional soil physicochemical properties is Kriging spatial interpolation. It is a spatial interpolation method based on the unbiased optimal estimation of the values of regionalized variables in a finite area based on a variational function model [18]. Based on the theory of regionalized variables, the structural nature of the original data and the semi-variance function is used to calculate the linearly weighted combination of the accessible variables, so as to provide an unbiased optimal estimation of the predicted values at the point to be estimated, and this method is capable of calculating the degree of reliability of the estimated values (the variance of the estimated values). In this paper, three Kriging interpolation methods (Ordinary Kriging, Simple Kriging and Pan Kriging) are used for spatial interpolation. A detailed description of the three methods is provided in section 1.1 of the supplementary materials in S1 File.

### 2.6 Predictive accuracy assessment

The accuracy of the results of the study was tested by comparing the predicted values of different interpolation methods for different fitted models with the sampled measured values through cross-validation using mean error (ME), mean absolute error (MAE), and root mean square error (RMSE) Evaluating the accuracy of interpolation methods (H and M, 1989). For the specific calculation formula, please refer to supplementary Materials 1.2 in S1 File.

### 2.7 Statistical analysis

All spatial interpolation procedures, including variogram fitting and Kriging simulations (OK, SK, UK), were conducted using ArcGIS 10.8 (Esri, Redlands, CA, USA) and verified in GS + 10.0 for semivariogram optimization. Statistical analyses and figure generation were performed in OriginPro 2021 and SPSS 26.0.

## 3. Results and analysis

### 3.1. Distribution characteristics of organic carbon in different types of soils

As shown in Table 1, the average organic carbon content under different land use modes was in the following order from high to low: Sediment (141.11)> swamp (34.37)> grassland (30.80)> agricultural land (27.05)> forest land (15.10). From the analysis of the coefficient of variation, it can be seen that the marsh and grassland have high intensity variability, and the bottom mud, forest land and agricultural land have medium intensity variability.

**Table 1:** Statistical analysis of soil organic carbon (SOC) content across different land use types in Caohai Wetland.

Soil type	Content range(g/kg)	Average content(g/kg)	Standard deviation	Coefficient of variation	Skewness factor	Kurtosis coefficient
Sediment	52.73-241.06	141.11	60.03	0.43	0.40	−1.29
Swamp land	12.33-65.41	34.37	23.70	0.69	0.82	−0.74
Grasslands	13.17-81.47	30.80	24.14	0.78	1.94	3.95
Woodland	5.54-20.41	15.10	6.12	0.41	−1.15	0.58
Agricultural land	14.68-44.45	27.05	8.09	0.30	0.36	−0.70

**Table 2:** Semi-variational model and fitting parameters for soil organic carbon content.

Interpolation method	Fit model	Nugget value	Abutment value	Stump base ratio	Variable range/km	Average error	Standard error of the mean	Rms error
Ordinary Kriging	Gaussian function	441.196	3629.615	0.122	0.033	−0.525	26.932	26.502
	hole effect	448.779	2926.232	0.153	0.045	−0.733	25.985	26.897
	K-Bessel	435.651	3693.140	0.118	0.035	−0.464	27.198	26.485
	J-Bessel	451.458	2596.738	0.174	0.046	−0.356	25.780	27.631
Simple Kriging	Gaussian function	414.083	4449.363	0.093	0.037	−0.542	25.621	26.533
	hole effect	367.693	3018.896	0.122	0.037	−0.819	25.086	28.439
	K-Bessel	408.526	4284.957	0.095	0.037	−0.478	26.280	26.523
	J-Bessel	359.578	3391.709	0.106	0.037	−0.624	25.897	27.519
Universal kriging	Gaussian function	595.771	441.260	1.350	0.026	−0.530	27.494	30.841
	hole effect	626.451	348.853	1.796	0.042	−0.695	27.557	31.656
	K-Bessel	519.010	539.436	0.962	0.003	−0.273	27.342	30.712
	J-Bessel	627.442	384.252	1.633	0.042	−0.623	27.586	31.762

SOC contents are significantly different of different soil types, and the land use type has a significant effect on soil organic carbon content (Fig 2). Subsoil is more concentrated in the whole study area, and its organic carbon content is the highest, which may be due to the distribution of subsoil in the watershed, the biological residues of the water body and plankton constantly deposited on the bottom of the water, so its organic carbon content is higher; the distribution of marsh in the figure is more extensive, dispersed along the center of the high-value area to the surrounding area, and its organic carbon content is higher, ranging from 12.33–65.41 g/kg, second only to the organic carbon content of the substrate; grassland is less distributed and dispersed throughout the study area, mainly located around the central substrate area, with a medium organic carbon content ranging from 13.17–81.47 g/kg; agricultural land and forest land are more dispersed, mainly at the boundary of the study area, with a low organic carbon content, which may be attributed to the fact that the agricultural land is continuously plowed and subject to. This may be due to the fact that agricultural land is continuously cultivated and subject to anthropogenic influences on the input and output of organic carbon, resulting in a loose soil structure and therefore low nutrient and organic carbon content.

### 3.2 Semi-variate fitting of soil organic carbon content

The semi-variational function model is the key to spatial description and prediction in geo-statistics. In this study, the semi-variational model was used to optimally fit the soil organic carbon content, and three different interpolation methods, OK, SK and UK, were used, which contained different fitting models, and by comparing a variety of parameters such as block-gold value, base-table value, and block-base ratio, it comprehensively reflected the performance of the different interpolation methods and the spatial characteristics of the soil organic carbon content.

The range of variation indicates the magnitude of the spatial autocorrelation range of the attribute factor, and is related to the scale of observation and the various ecological processes that affect the attribute factor at that scale. As can be seen from Table 2, J-Bessel has the largest range of variation, followed by hole effect, Gaussian function, and K-Bessel has the smallest. This indicates that when studying the spatial variability pattern of soil texture in this region, the sampling spacing can be appropriately reduced by using K-Bessel and increased by using J-Bessel. The block-base ratio indicates the proportion of spatial variability caused by random factors to the total variability of the system, and a high ratio indicates that random factors play a major role in causing the degree of spatial heterogeneity, and vice versa indicates that the variability caused by structural factors plays a major role. The results show that the block base ratios of ordinary kriging range from 0.118 to 0.174, those of simple kriging range from 0.095 to 0.122, and those of pan kriging are relatively high, ranging from 0.962 to 1.796. This indicates that both the OK and SK methods exhibit strong spatial autocorrelation, except for the UK method.

In order to compare the different interpolation methods, when determining the fitting model as Gaussian function, three interpolation methods of OK, SK and UK are selected for interpolation, comparing the prediction errors of the three methods, the average errors of the three methods are not much different in general, the SK method is the smallest, and the UK method is the largest, and the difference between the remaining two models is not significant. Therefore, from the results of error analysis, the SK method is better than the other methods; when determining the fitting model for the hole effect, the three methods have their own advantages and disadvantages, with no obvious distinction; when determining the fitting model for the K-Bessel, the average standard error and the root mean square error of the SK method are smaller, so the SK method is better than the others; when determining the fitting model for the J-Bessel, the average error is minimized by the OK method, and the UK and SK methods are the second largest. In determining the fitting model as J-Bessel, the average error is minimized by OK method, followed by UK and SK methods; the average standard error is minimized by OK method, followed by the rest; the root mean square error of OK and SK methods is close to and minimized, so from the point of view of error analysis, OK method is better than other methods. Overall, the OK method and the SK method are better.

### 3.3. Prediction of soil organic carbon content based on different interpolation methods

**Fig 3:**
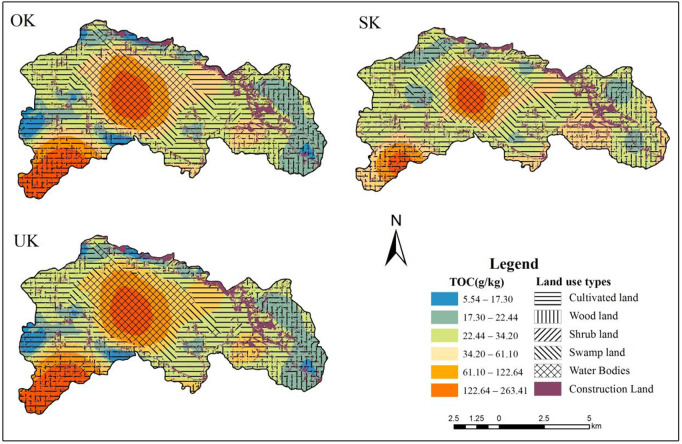
Predicted spatial distribution of soil organic carbon based on different interpolation methods.

The spatial distribution of soil properties is both random and correlated, and classical statistics cannot explain its spatial distribution and variance structure. With the introduction of Kriging interpolation into the field of soil research, the study of spatial characteristics of soil properties has been simplified. To understand the spatial characteristics of soil particle composition more intuitively, this study plotted the spatial distribution of soil organic carbon under different interpolation methods (Fig 3). The results show that soil organic carbon under three different interpolation methods has similar spatial distribution characteristics. As can be seen from Fig 3, the soil organic carbon content characteristics showed a combination of ring and strip distribution in the sampling area. The organic carbon content was higher in the middle part of the sampling area and decreased in the north-south direction, respectively. In the southwest corner of the sampling area, the organic carbon content was higher, with values ranging from 34.20 to 263.42 g/kg.

Comparing the results of the three interpolation methods, the SK method in Fig. 3 is slightly better than the OK method in local details. This may be due to the fact that ordinary kriging (OK) interpolation only considers the spatial information of soil organic carbon itself, whereas simple kriging (SK) interpolation is able to utilize certain variables with better correlation with soil organic carbon to improve the accuracy of the valuation of soil organic carbon content. In addition, the spatial distribution structure presented by the UK method has stronger spatial randomness and heterogeneity. In terms of local structure, the UK method can better reflect the randomness and dependence of the spatial distribution of soil organic carbon content, and its simulation effect is more realistic and applicable compared with the OK and SK interpolation methods.

From the spatial interpolation results of soil organic carbon content in Table 3, the predicted values of different interpolation methods for different soil types (subsoil, marsh, grassland, forest land and agricultural land) were different: the predicted values of OK and SK for subsoil were close to each other, while the predicted value of UK for subsoil was 121.25, which was different from that of the previous two methods; the predicted values of OK and SK for marsh were close to each other, while the predicted value of UK was higher; the predicted values of all three interpolation methods for grassland were close to each other, indicating that the predicted results of grassland were relatively consistent under different methods. The predicted values of the three interpolation methods for grassland were close to each other, indicating that the predicted organic carbon content of grassland was relatively consistent under different methods. The results further illustrate the characteristics and applicability of different interpolation methods in predicting the spatial distribution of soil organic carbon content.

**Table 3:** Results of spatial interpolation prediction of soil organic carbon content.

Soil type		Sediment	Swamp land	Grasslands	Woodland	Agricultural land
Interpolation method	OK	134.30	27.15	29.66	27.09	28.55
	SK	134.18	27.19	30.11	26.71	28.82
	UK	121.25	29.02	30.11	33.81	32.36

### 3.4 Optimal interpolation method for soil organic carbon content prediction

**Fig 4:**
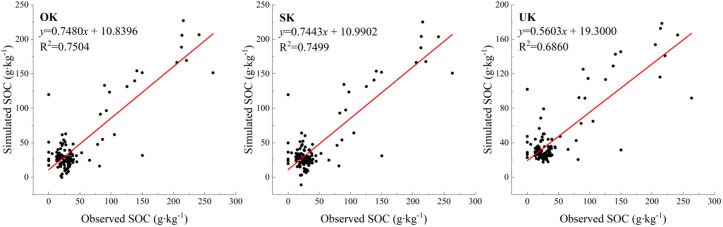
Comparison of measured and predicted values of different interpolation methods.

The spatial distribution of soil organic carbon under the three different interpolation methods was fitted with the optimal interpolation method to obtain the predicted values of the different interpolation methods, and scatter plots were made with the measured values as the horizontal coordinates and the predicted values as the vertical coordinates (Fig 4). Add the trend line in the scatter plot, obviously, when the trend line is closer to the straight line in the 45° direction, it means that the predicted value is closer to the measured value and the interpolation result is more ideal. Comparing the coefficient of determination R of the three interpolation methods, the larger the value of R is, the higher the accuracy of spatial interpolation, and it can be seen from the figure that the coefficients of determination are in the order of: OK > SK > UK, which indicates that the predicted value of the OK method has a better fit with the measured value, followed by SK, and the worst of the UK method.

The prediction accuracy statistics of different spatial interpolation methods are shown in Table 4. Ordinary Kriging (OK) and Simple Kriging (SK) achieved the lowest RMSE (2.41) and identical MAE (0.14), with R² values of 0.913 and 0.911 respectively. In contrast, Universal Kriging (UK) had a higher RMSE (2.80) and lower R² (0.863). These values demonstrate that OK and SK outperformed UK in predictive stability and overall accuracy.

**Table 4:** Prediction accuracy of different spatial interpolation methods.

Interpolation method	Average error	Standard error of the mean (statistics)	Rms error	Improved accuracy (%)
OK	0.14	0.22	2.41	13.77
SK	0.14	0.22	2.41	14.17
UK	0.15	0.25	2.80	32.48

From Table 4, it can be seen that the mean error and mean standard error are the same as well as the root-mean-square error for the OK and SK methods, but the improved accuracy of the SK method (14.17%) is slightly higher than that of the OK method (13.77%); and the mean error, mean standard error, and root-mean-square error for the UK method are larger than those of the OK and SK methods, but its improved accuracy (32.48%) is significantly higher than the first two. The SK method achieved a marginally higher accuracy improvement rate (14.17%) than OK (13.77%), a 0.4% relative advantage. while the UK method performs poorly in terms of the coefficient of determination but excels in terms of improving accuracy. If the degree of fitting is more important, then the OK method is optimal; if the improvement of accuracy is more important, then the UK method is optimal. The specific choice of method needs to be decided according to the actual research needs and the focus on different indicators.

### 3.5 Influencing factors and dominant factors of soil organic carbon

Soil organic carbon (SOC) is an essential component of the soil solid phase, serving as a critical nutrient source alongside soil minerals. A variety of physical, chemical, and biological soil properties exert both direct and indirect influences on SOC content. In this study, correlation analysis was conducted between SOC and 15 soil physicochemical factors. As shown in Table 5, SOC exhibited significant correlations with most of these variables. SOC showed highly significant positive correlations with total nitrogen (TN), available nitrogen (alkali-hydrolyzed nitrogen, AN), Hg, Cd, As, Pb, Zn, DDT, and OCPs. The order of correlation strength was: AN (0.936)> TN (0.925)> Cd (0.813)> Zn (0.749)> DDT (0.698)> OCPs (0.652)> Hg (0.470)> As (0.456)> Pb (0.362). In addition, SOC was significantly positively correlated with HCE (0.352), and strongly negatively correlated with pH (−0.694). No significant correlations were found with total phosphorus (TP), available phosphorus (AP), Cr, or Cu.

**Table 5:** Correlation analysis of factors affecting soil organic carbon content.

impact factor	correlation coefficient	impact factor	correlation coefficient	impact factor	correlation coefficient
pH	−0.694^**^	Hg	0.47^**^	Cu	0.226
TN	0.925^**^	Cd	0.813^**^	Zn	0.749^**^
TP	0.125	As	0.456^**^	HCE	0.352^*^
AN	0.936^**^	Pb	0.362^**^	DDT	0.698^**^
AP	0.082	Cr	0.135	OCPs	0.652^**^

Note:

** indicates that the correlation is highly significant at a confidence level (double test) of 0.01;

* indicates that the correlation is significant at a confidence level (double test) of 0.05. TN, Total nitrogen; TP, Total phosphorus; AN, available nitrogen; AP, Available Phosphorus; HCE, Hexachlorocyclohexane; DDT, Dichlorodiphenyltrichloroethane; OCPs, Organochlorine Pesticides.

**Fig 5:**
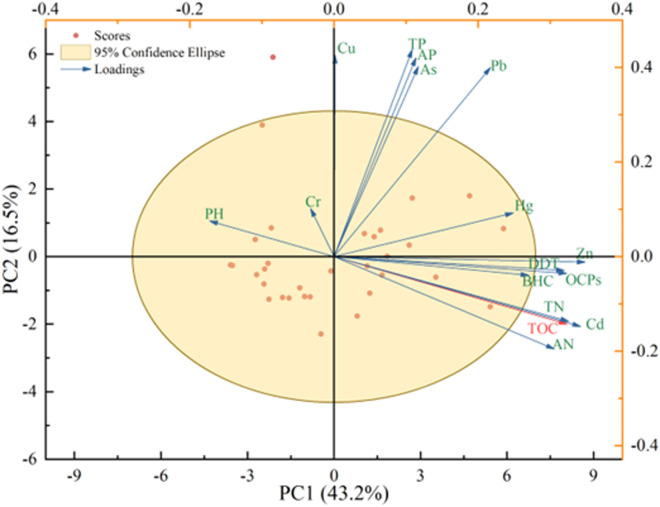
Principal component analysis of soil organic carbon content.

Principal component analysis (PCA) was used to further determine the major factors affecting SOC. Two principal components with eigenvalues greater than 1 were extracted, explaining 81.3% of the total variance. The first component (PC1) accounted for 56.3% of the variance and had strong positive loadings for TN, AN, Cd, Zn, DDT, and OCPs, indicating that these factors are the dominant sources of variation in SOC content and reflect strong anthropogenic and agricultural influence. The second component (PC2) explained 25.0% of the variance and showed a strong negative loading for pH, confirming its inverse relationship with SOC. The spatial relationships and variable contributions to PC1 and PC2 are illustrated in Fig 5, where loading vectors distinctly separate nutrient- and pollution-related variables from pH, confirming the dual-axis control structure of SOC dynamics in the wetland soils.

## 4. Discussions

### 4.1 Differences in organic carbon content in different types of soils in the Caohai

This study reveals that the spatial distribution of soil organic carbon in the Caohai Wetland is significantly correlated with land use types. High-value areas were mainly concentrated in the sediment in the central part of the water body, which was attributed to the continuous deposition of biological residues and plankton in the water body, resulting in the continuous accumulation of organic carbon in the sediment [19,20]. In addition, high values of organic carbon were also found in the southwestern part of the water body, which was dominated by agricultural and forested land with a small amount of grassland, contrasting with the soil organic carbon content of the agricultural land around the water body of the Sea of Grass. This difference may be due to the intensity of human activities in different regions, because the level of soil organic carbon in agricultural soils is affected by a variety of factors, including land management practices, land use, soil texture, sediment source, moisture content, topography, and vegetation cover type [21].

We note that land use change is known to impact SOC distribution by altering input sources and microbial dynamics. Although our study did not include direct LULC map overlays, spatial patterns in SOC concentration correspond with historical land conversion data from Caohai. We recommend integrating remote sensing–derived land use transitions in future SOC modeling to enhance explanatory power [22,23].

### 4.2. Broader implications for Spatial Modeling and SOC Management

Our findings on Kriging interpolation performance provide valuable insights for spatial modeling of soil organic carbon (SOC) in complex wetland environments characterized by strong spatial heterogeneity [24]. Accurate SOC mapping is essential for wetland conservation planning, carbon budgeting, and ecological restoration. In particular, identifying zones of high SOC concentration can inform targeted land management, erosion control, and climate mitigation strategies.

This study demonstrates that the combination of Ordinary Kriging (OK) with Bessel-type variogram models (J-Bessel and K-Bessel) achieves higher prediction accuracy in heterogeneous landscapes compared to conventional methods such as inverse distance weighting (IDW), trend surface analysis, or even Universal Kriging with basic variograms. The J-Bessel and K-Bessel models outperformed others in capturing local SOC variability because of their oscillatory nature, which allows better fitting of complex spatial structures [25]. These models are particularly effective in heterogeneous wetland settings where SOC values fluctuate over short distances due to mixed land use and microtopography. Compared to smooth variograms like the Gaussian model, Bessel-type models offer higher flexibility in adapting to patchy patterns, leading to lower RMSE (e.g., 2.41 for OK + J-Bessel vs. 2.61 for OK+ Gaussian) and improved R² (0.913 vs. 0.901).

Furthermore, when comparing predictive performance across models, OK and SK methods both achieved lower mean errors (0.14) and RMSE values (2.41) than UK (RMSE = 2.80), while also producing higher R² values (OK: 0.913; SK: 0.911; UK: 0.863). These quantifiable improvements demonstrate that Bessel-based OK and SK models outperform others in predictive stability and model fitting accuracy.

These findings suggest that customizing the variogram structure to reflect actual landscape complexity is more important than simply choosing an interpolation method. The spatial autocorrelation captured by Bessel functions enables more accurate SOC surface simulations in fragmented ecosystems. Therefore, integrating variogram selection with land use zoning and soil unit boundaries can optimize mapping frameworks for future studies. Additional spatial covariates, such as LULC change layers, vegetation indices, and hydrological conditions, should be incorporated in follow-up models to enhance SOC prediction under dynamic environmental conditions.

## 5. Conclusions

Organic carbon estimation using relatively simple, accurate and inexpensive geophysical methods combined with advanced statistical techniques for spatialization of organic carbon can provide a feasible solution for studying the sustainability of wetland ecosystems. When predicting and modeling the organic carbon distribution of soils in plateau wetland areas with strong spatial heterogeneity, the choice of interpolation methods and fitting models has a great influence on the prediction and simulation results, and this issue should be brought to the attention of scholars. In this study, the OK method performed optimally in an integrated manner and provided effective methodological support for the study of the spatial distribution of soil organic carbon in the Caohai wetland of Guizhou. It provides a methodological reference for soil organic prediction in the plateau wetland areas of China, and even in other areas with similar spatial heterogeneity.

## Supporting information

S1 File Supplementary materials.docx
(DOCX)
