# Supplementary material for: Comparative analysis of soil organic carbon across different land types in plateau wetlands using Kriging interpolation based on spatial heterogeneity
Source: PLoS One. 2025 Jul 23;20(7):e0328246. doi: 10.1371/journal.pone.0328246 (PMC12286344; doi:10.1371/journal.pone.0328246)
Supplement: S1 File — Supplementary materials.docx (DOCX) [file pone.0328246.s001.docx]

**Supplementary materials**

**Comparative analysis of soil organic carbon across different land types in plateau wetlands using Kriging interpolation based on spatial heterogeneity**

**1.1 Three different interpolation methods**

Ordinary Kriging (OK), which works on the assumption that a constant mean value is unknown throughout the process, uses neighborhood known data at unsampled locations to estimate values using interpolation (H and Kriging, 2003). The calculation of OK is shown (Asa et al., 2012) as follows:

$$Z_{OK}^{*}(x)=\sum_{i=1}^{n} \omega_{i}(x)\cdot Z(x_{i})+\left[1-\sum_{i=1}^{n} \omega_{i}(x)\right]m(x)$$

$Z_{OK}^{*}\left(x\right)$Simple Kriging (SK) method assumes that the mean of the smooth random variable is constant and known. Therefore, mathematically, it is the simplest but least general method. Simple Kriging SK calculation is shown (Asa et al., 2012) :

$$Z_{SK}^{*}(x)=\sum_{i=1}^{n} \omega_{i}(x)\cdot Z(x_{i})+\left[1-\sum_{i=1}^{n} \omega_{i}(x)\right]m$$

In equations (1) and (2), where *Z*(*x*)= random variable at the location *x*; all *x_i_* values = *n* data locations; *m*(*x*)= *E*｛*Z*(*x*)｝= location-dependent expected value of the *RV* *Z*(*x*) ; $Z_{OK}^{*}(x)$or$Z_{SK}^{*}(x)$= linear regression estimator; *ω_i_*= weights; and *m*(*x*)= mean.

In ordinary and simple kriging, the mean of a variable is assumed to be constant over the search area (local smoothness). In some practical cases, the local mean is different over the search area. Pan-Kriging (Universal Kriging, UK) deals with the case where the local mean is variable over the study area. Universal Kriging is designed to accommodate a non-smooth mean where the expectation of *Z*(*x*) is a deterministic function of the coordinates. The stochastic function *Z*(*x*) is a combination of the trend component with deterministic variation *m*(*x*) and the residual component *R*(*x*) with μstochastic or random variation, the UK calculations are shown as follows (Asa et al., 2012) :

Where *λ_k_*(*x*) = known functions of the coordinates or the known basis function; and μ_k_= fixed, unknown coefficients/parameters

$$Z_{UK}^{*}(x)=\sum_{i=1}^{n} \omega_{i}(x)\cdot Z(x_{i})$$

The residual component, *R*(*x*), is considered a stationary random variable with zero mean and a covariance *C_R_*(*h*).

Kriging weights are generally calculated based on the distance between the observation and the target location; the closer the observation is to the point of interest, the greater the weight. It assumes the concept of smoothness, which means that the mean and variance are constant throughout the spatial field.

**1.2 Calculation formula for accuracy assessment**

ME and MAE determine the degree of bias obtained in the prediction. Higher values indicate a greater discrepancy between predicted and measured values (Varouchakis and Hristopulos, 2013) The calculation of ME and MAE are shown as follows:

The root mean square error RMSE is actually the sum of the squares of the deviations of the observations from the true value and then the number of observations N to find the ratio, and then the square root to get the calculation.

Root Mean Square Error (RMSE) is used to quantify the accuracy of the interpolation model used.The lower the RMSE value, the higher the reliability of the model. The Root Mean Square Error (RMSE) is calculated as follows：

$$RMSE=\sqrt{\frac{1}{N}\sum_{i=1}^{N} \left(x_{oi}-x_{pi}\right)^{2}}$$

The *N* all denote the total number of validation points, and *x_oi_* denotes the experimental value of the validation points, and *x_pi_* denotes the estimation model.

Table S1 Variational function model expressions (Pasini et al., 2014)

| Model | Functional expression |
| --- | --- |
| Gaussian | $\gamma\left(h\right)=C_{0}+C_{1}\left[1-\text{e}^{-3\left(\frac{h}{a}\right)^{2}}\right]$ |
| Hole Effect | $\gamma\left(h\right)=C_{0}+C_{1}\frac{1-\sin\left(\frac{2\pi h}{a}\right)}{\sin\left(\frac{2\pi h}{a}\right)}$ |
| K-Bessel | $\gamma\left(h\right)=C_{0}+C_{1}\left[1-\frac{\left(\frac{\Omega_{\theta_{k}}h}{a}\right)^{\theta_{k}}}{2^{\theta_{k^{-1}}}\Gamma\left(\theta_{k}\right)}K_{\theta_{k}}\left(\frac{\Omega_{\theta_{k}}h}{a}\right)\right]$ |
| J-Bessel | $\gamma\left(h\right)=C_{0}+C_{1}\left[1-\frac{2^{\theta_{d}}\Gamma\left(\theta_{d}+1\right)}{\left(\frac{\Omega_{\theta_{d}}h}{a}\right)^{\theta_{d}}}J_{\theta_{d}}\left(\frac{\Omega_{\theta_{d}}h}{a}\right)\right]$ |

**References**

Asa, E., Saafi, M., Membah, J., Billa, A., 2012. Comparison of Linear and Nonlinear Kriging Methods for Characterization and Interpolation of Soil Data. J. Comput. Civil. Eng. 26(1), 11-18.

H, W., Kriging, O., 2003. in Multivariate Geostatistics. Switzerland.

Pasini, M.P.B., Lúcio, A.D., Cargnelutti, A., 2014. Semivariogram models for estimating fig fly population density throughout the year. pesqui. Agropecu. Bras. 49(7), 493-505.

Varouchakis, E.A., Hristopulos, D.T., 2013. Comparison of stochastic and deterministic methods for mapping groundwater level spatial variability in sparsely monitored basins. Environmental Monitoring and Assessment 185(1), 1-19.
